# Using radiochemistry to diagnose fuel-ablator mix in inertial confinement fusion studies at the National Ignition Facility: measurement of Tc/Mo isotopic ratios for the Pushered Single Shell campaign

**DOI:** 10.3389/fchem.2025.1632948

**Published:** 2025-09-08

**Authors:** Kelly N. Kmak, John D. Despotopulos, Tony L. Huynh, Brian N. Sammis, Keenan Thomas, Todd Wooddy, Eduard L. Dewald, Stephan A. MacLaren

**Affiliations:** Lawrence Livermore National Laboratory, Livermore, CA, United States

**Keywords:** National Ignition Facility, inertial confinement fusion, radiochemical separations, Mo, Tc

## Abstract

The development of radiochemical measurement techniques as a diagnostic for fusion experiments at the National Ignition Facility enables a new method for assessing fuel-ablator mix and the impact of this mix on capsule performance. Diagnosing capsule mix in internal confinement fusion studies is difficult due to the small spatial scales (10s of µm) and short-time frames (100s of ps) over which the mix typically evolves in these experiments. For the Pushered Single Shell campaign, radiochemical measurements on debris collected from fusion experiments can be used to determine isotopic ratios of activation products, particularly ^96g^Tc/^99^Mo and ^95g^Tc/^99^Mo, to provide vital information on nuclear reactions in the burning plasma that can inform simulations that seek to understand the degree of capsule-fuel mix and the impact on the capsule performance. These radiochemical measurements have been conducted regularly since November 2023 providing data on a range of capsule designs and neutron yields. Data from eight NIF experiments is presented, the measured ^96g^Tc/^99^Mo and ^95g^Tc/^99^Mo range from (0.5–5) × 10^–4^ to (0.3–3) × 10^–4^, respectively. The development of radiochemical diagnostics aids in understanding and optimizing the design of fusion experiments, providing unique and valuable insights into capsule behavior and directly measuring fuel-ablator mix.

## 1 Introduction

The National Ignition Facility (NIF) at Lawrence Livermore National Laboratory (LLNL) is a high average power laser used for inertial confinement fusion (ICF) studies (Spaeth et al., 2016). In these experiments, fusion is induced in a small capsule (∼1 mm radius) via an indirect drive mechanism wherein 192 laser beams are focused onto the inner surface of a high-Z “hohlraum” creating x-rays that drive the heating and compression of the deuterium-tritium (DT)-filled capsule through ablation of the outer layer of the capsule (Dewald et al., 2024; Zylstra et al., 2019). There are various capsule designs for different fusion experiments with specific parameters to optimize the plasma conditions (Dewald et al., 2022; Dewald et al., 2024).

One viable fusion capsule design is the Pushered Single Shell (PSS), which incorporates a high-Z metal (Cr, Mo) into a Be ablator. Using a gradient of a high-Z metal dopant into the low-Z shell can offer improvements for radiative losses and tamping to enhance the fuel performance during the fusion reaction. This design has been extensively described by E. Dewald and S. MacLaren both experimentally (Dewald et al., 2022; Dewald et al., 2024) and through simulations (MacLaren et al., 2021). A critical component of the success of this design is minimizing the amount of high-Z material that mixes into the burning plasma as this decreases the plasma temperature and thus the yield (Dewald et al., 2022). This is accomplished by fabricating the capsule such that the density is increased gradually from the ablator to the pusher layer to minimize hydrodynamic instabilities by increasing the density gradient scale length. Determining the extent of high-Z mix that occurs during these ICF experiments is an important parameter to understand and optimize capsule design.

A demonstrated method for diagnosing the amount of mix is by using radiochemical signatures. This has been described for PSS capsules based on Cr (MacLaren et al., 2025). Briefly, as the fusion reaction occurs in a region of high temperature and pressure at the center of the capsule implosion, both energetic protons and deuterons are produced alongside neutrons. The charged particles have a much shorter range than the neutrons and primarily undergo nuclear reactions in the regions of high mix where the increased electron density from the pusher ions significantly reduces the range of these particles. Neutrons have a much longer range and interact with all areas of the capsule, not just those in contact with the fuel (MacLaren et al., 2025). Therefore, comparing the number of atoms of a nuclide produced via charged particle reactions, indicative of capsule mix, versus a radionuclide produced via neutron reactions gives a metric to identify the degree of capsule mix normalized by the yield. In the case of Cr capsules, the ratio used as a mix diagnostic was ^52^Mn/^51^Cr. In this case, ^52^Mn arises from (p,n) and (d,2n) reactions on ^52^Cr, the dominant stable isotope (83.8%) of chromium (Rosman and Taylor, 1998), and ^51^Cr is produced from (n,2n) reactions on ^52^Cr (MacLaren et al., 2025). The results of these experiments and comparison to simulations are described in MacLaren et al. (2025).

**FIGURE 1:**
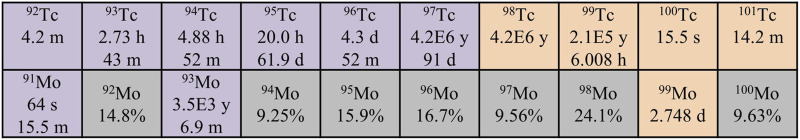
Isotopes of Tc and Mo. Stable isotopes, and their isotopic abundances, are indicated in grey (Rosman and Taylor, 1998). Radioactive isotopes are given with their half-lives (NNDC, 2023); the half-life of the ground state is listed first, followed by the half-life of a metastable state, if relevant. Isotopes in purple decay by electron capture or β^+^; isotopes in orange decay by β^−^. Decay via isomeric transition is not indicated for simplicity.

The Cr capsules served as surrogates for the optimization of the implosion characteristics during the development of Mo capsules, which are predicted to provide improved implosion characteristics (Dewald et al., 2022). The shift from Cr to Mo required the identification of new isotopes to provide a mix diagnostic as well as the development of radiochemical techniques to quantify these isotopes. Natural Mo has several stable isotopes (see Figure 1). As the primary neutron reaction from the 14.1 MeV DT fusion neutrons is (n,2n), the possible candidates for radioactive Mo isotopes are: ^91^Mo, ^93^Mo and ^99^Mo. Of these isotopes, only ^99^Mo has a half-life suitable for analysis over a ∼1 day period (Figure 1). Proton and deuteron reactions on ^nat^Mo can make a wide range of Tc isotopes, however, only ^95g^Tc and ^96g^Tc have half-lives favorable for analysis. This leads to two possibilities for radiochemical mix diagnostics with Mo capsules: ^95g^Tc/^99^Mo and ^96g^Tc/^99^Mo.

When a NIF shot occurs, the large amount of radiation produced prohibits the measurement of the reaction products of interest *in situ*. Instead, Solid Radiochemistry Collectors (SRCs) are fielded in the NIF target chamber; they collect solid debris from the implosion as it condenses and are analyzed post-shot. The use of SRCs has been extensively described, see Refs. Despotopulos et al. (2022) and Despotopulos and Shaughnessy (2024). For the purposes of PSS experiments, SRCs are vanadium discs fielded at specific positions on a Diagnostic Instrument Manipulator (DIM) in the NIF chamber. The DIM positions relevant for the measurement of radiochemical signatures are 90–315 and 90–78, though in practice only 90–315 is used routinely. The positions of DIMs in the NIF chamber are labeled with a modified polar coordinate system (polar angle, azimuthal angle), therefore both DIMs 90–315 and 90–78 are in the equatorial plane, as is the capsule and hohlraum which sit at the target chamber center (TCC).

The SRCs are very radioactive post-shot as they collect debris not only from the capsule, including the reaction products of interest, but also from the hohlraum which contains Au and depleted-U. The activation products from Au as well as both activation and fission products from U create a high gamma background on the SRCs over which the relatively small signal from the Tc isotopes cannot be detected. Chemical processing is essential to separate the Tc isotopes from the SRCs to enable measurements of the activity of these isotopes. There are considerable challenges for the chemical separations for these experiments. The short half-life of ^95g^Tc necessitates rapid chemical separations, ideally with no more than one half-life (20 h) elapsing between the shot and the nuclear decay counting. Furthermore, the separations must have high yields and sufficiently high radiopurity for precise gamma spectroscopy measurements on low activity levels (∼0.2–2 decays per second (dps) of ^95g^Tc and ^96g^Tc. By measuring a ratio of two isotopes based on the individual SRCs, determination of the absolute collection efficiency is not required, only that the two isotopes can be measured from the same sample. This allows for comparison among different experiments without needing to directly account for variations in collection.

Using radiochemical signatures provides a unique diagnostic for ICF experiments as it is a direct measurement of fuel-ablator mix, which is not possible for metal-shell ICF capsules with any other diagnostic. Furthermore, these measurements are independent of any other NIF diagnostic system, are expected to be line-of-sight independent, and less affected by the implosion symmetry (MacLaren et al., 2025). The PSS campaign at NIF has been performing implosion experiments with Mo capsules since July 2023, N230708 in the NIF notation system (N Year-Month-Day), and the ^95g^Tc/^99^Mo and ^96g^Tc/^99^Mo isotope ratios have been reported routinely for these experiments since November 2023 with plans to continue for several years. The application of such measurements to benchmark mix models is described in MacLaren et al. (2025), this work only concerns the measurements themselves.

## 2 Materials and methods

### 2.1 Materials

All chemical processing was performed with ULTREX II ultra-pure acids (J.T. Baker). Aristar ultra-pure water (VWR International) was used for dilutions, as needed. The concentrated HNO_3_ had a concentration of 15–15.33 M as the concentrated varies slightly by batch. The SRCs were vanadium (99.8%, Goodfellow) fabricated, and polished under clean room conditions, by Pleasanton Tool & Mfg. Inc. into 2-inch (diameter) circles with a thickness of 0.5-mm. The ^96m^Tc tracer used in the chemical processing was produced based on the procedure in Kmak et al. (2024).

### 2.2 Activity measurements

All nuclear decay measurements were performed with high purity germanium (HPGe) detectors at the Nuclear Facility (NCF) at LLNL. All spectra collected from these experiments were analyzed with GAMANAL (Gunnink and Niday, 1972), which performs peak fitting on gamma spectra and uses a multi-line analysis to identify isotopes by their characteristic gamma-ray emissions. For ^99^Mo this included the six highest intensity gamma-ray emissions (Table 1). It should be noted that only the gamma emission from the direct decay of ^99^Mo are used in the analysis. While the short-lived daughter of ^99^Mo, ^99m^Tc, is sometimes used to identify ^99^Mo due to its strong gamma-ray emission at 140.5 keV (NNDC, 2023), it is not used in the analysis for these experiments as equilibrium between the two isotopes cannot be assumed. The gamma-ray emissions used to assess the Tc isotopes are listed in Table 1 as well. The high intensity 778.22 keV (99.76%) line from ^96g^Tc is not used in the analysis. This is to bar against any interference between any ^99^Mo (777.9 keV) and ^96g^Tc in case there is any residual ^99^Mo in the Tc samples.

**TABLE 1:** Nuclear decay data for relevant Mo and Tc isotopes (NNDC, 2023).

Isotope	Gamma-ray emission and intensities
^99^Mo	181.1 keV (6.05%) 366.4 keV (1.20%) 528.8 keV (0.0532%) 739.5 keV (12.2%) 777.9 keV (4.31%) 823.0 keV (0.134%)
^95g^Tc	765.8 (93.8%)
^95m^Tc	204.1 keV (63.2%) 582.1 keV (30.0%) 820.6 keV (4.71%) 835.1 keV (26.6%)
^96g^Tc	812.5 keV (82%) 849.9 keV (98%)

### 2.3 SRC fielding and retrieval

**FIGURE 2:**
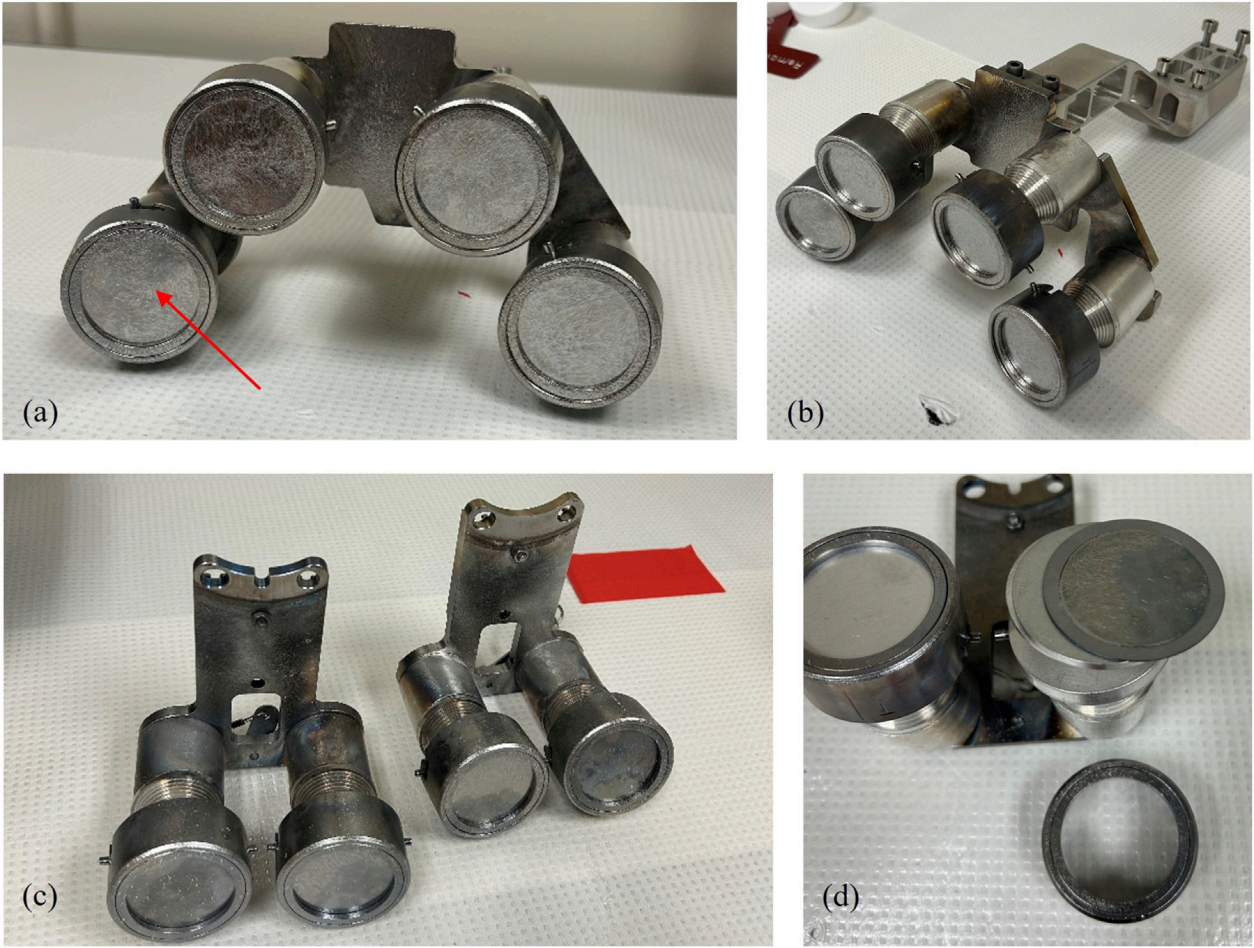
**(a)** The SRC bracket assembly for DIM 90–315 (front view) with a red arrow indicating the exposed face of one SRC; during the shot the bracket is oriented vertically (rotated 90° from the image). **(b)** A side view of the SRC assembly for 90–315. **(c)** The SRC brackets for 90–78; one piece goes above the DIM and the other below. **(d)** The SRC bracket with the front clamp removed and the SRC resting on the base.

For each PSS experiment at NIF, there were four SRCs fielded on DIM 90–315 with a standoff distance of 30 cm from TCC. Some experiments also had SRCs fielded on DIM 90–78 with a standoff distance of 32–50 cm. The brackets that hold the SRCs for both DIMs are shown in Figure 2. A maximum of two SRCs from 90–78 were analyzed for radiochemical signatures due to resource limitations, although four SRCs can be fielded on this DIM.

**FIGURE 3:**
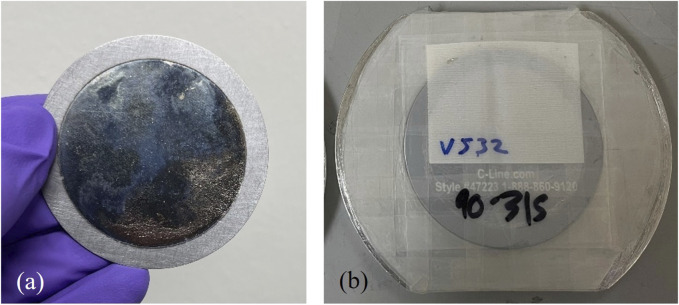
**(a)** Damage to an SRC surface from the implosion debris wind (the undamaged ring of material on the outsider edge is the area covered by the clamp during the experiment). **(b)** SRC mounted for gamma spectroscopy. The holder ensures the positioning of the SRC is replicable between experiments.

After each NIF shot was completed, the SRC brackets were removed from their positions in the NIF chamber and transported to the radiochemistry building at LLNL. Each SRC was removed from the bracket and mounted into an aluminum holder for initial characterization via gamma spectroscopy at the NCF (see Figure 3). The delay between the shot and the initial nuclear decay counting is typically ∼3–6 h. These counts were 4–12 h long depending on the level of activity as the goal is to achieve <3% counting uncertainty on ^99^Mo activity during this time.

### 2.4 Chemical processing

**FIGURE 4:**
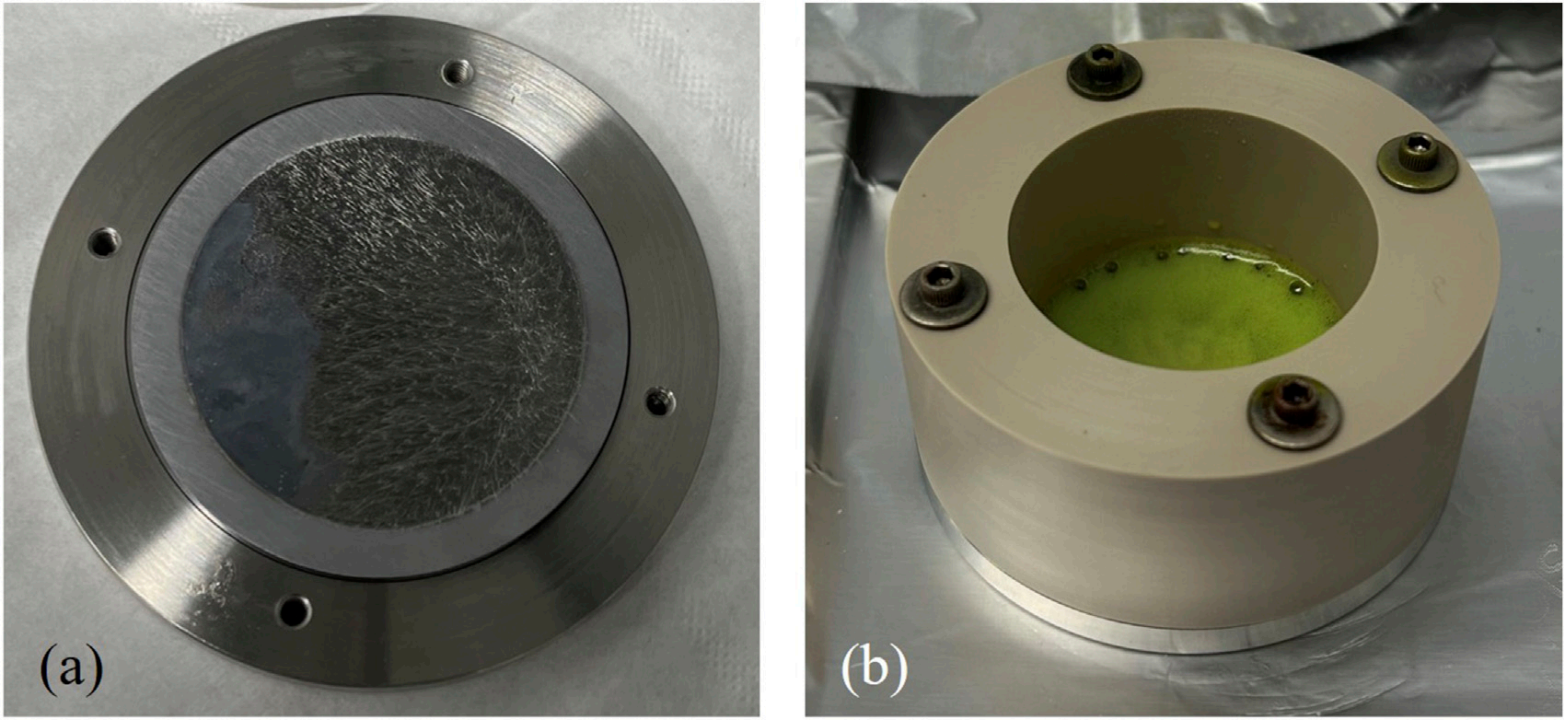
**(a)** An SRC in the base of the leaching cell. **(b)** An SRC during the leaching process in the fully assembled cell.

Once the initial counting was completed, the SRCs undergo chemical processing. First, each SRC was mounted in a leaching cell. The cells have a stainless-steel base and PEEK (polyether ether ketone) ring with a groove that holds the SRC in place (Figure 4); the two pieces are screwed together to create a liquid-tight seal. To leach each SRC, first the SRC in the leaching cell was heated to 120 °C on a hotplate for a minimum of 20 min to ensure the stainless-steel base and the SRC itself are hot. Then, 2 mL concentrated HCl and 100 µL concentrated HNO_3_ was added to the cell and allowed to react for 1–2 min. It was clear the leaching was completed when the solution stopped emitting brown gas and the color of the solution shifted from yellow-green to green-blue. The acid was then removed, and this process (adding acid, reacting, removal) was repeated five more times. All the leachate solutions for each SRC were combined into one 50 mL centrifuge tube; the final volume was 9–10 mL due to evaporative losses during the leaching process. Leach solutions generally have a concentration of ∼12 mg/mL of ^nat^V.

**FIGURE 5:**
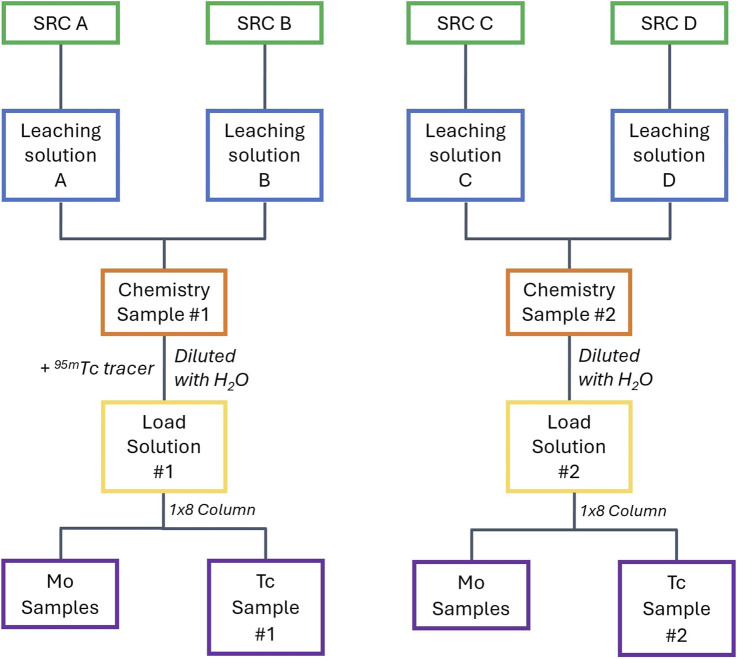
Chemical processing flow chart for four SRCs. This can be extrapolated to more SRCs in sets of two.

After all the SRCs have been leached, the leachate solutions were combined in sets of two to have two samples for further chemical separations (from the initial four SRCs, see Figure 5). Combining the leachate solutions increases the counting statistics in the final Tc samples, reducing uncertainty while ensuring more than one measurement per experiment for validation. After combining the leachate solutions, each sample was diluted to ∼6 M HCl (from ∼11 M HCl) and a 20 µL spike of ^95m^Tc in concentrated HNO_3_ was added to one sample to serve as a tracer for the column separation. Due to the secular equilibrium between ^95m^Tc and its shorter-lived ground state, ^95g^Tc, the tracer solution contained both these isotopes. Consequently, samples that contain the tracer cannot be used to identify the ^95g^Tc/^99^Mo ratio from the capsule as the ^95g^Tc from the tracer swamps the signal from the capsule. There was no detectable ^96m,g^Tc in the tracer solution.

A 5 mL Pierce Disposable Column (Thermo Scientific) with AG 1x8 resin (100–200 mesh, BioRad) was prepared for each chemistry sample and pre-conditioned with 20 mL 6 M HCl. Each of the diluted chemistry samples was loaded onto a column and fractions were collected as shown in Table 2. The columns can be run simultaneously for the two (or occasionally three) chemistry samples. After elution, each of the Mo samples (fractions 4–6) and the Tc sample (fraction 7) from each column were delivered to the NCF immediately for characterization with an HPGe detector. The Mo samples were counted for 4–8 h (depending on the activity) to achieve <3% uncertainty on the activity of ^99^Mo. The Tc samples were counted for 10 days, with a spectrum output every 2 days. The other chemistry samples were not counted for every experiment as the efficacy of separation has been established, but they can be analyzed for verification depending on detector availability. As with the Mo samples, they would be counted immediately after the chemistry for 4–8 h.

**TABLE 2:** The acids and volumes used in the column elution.

Fraction	Acid	Volume
Load Solution	∼6 M HCl, trace HNO_3_	20 mL
Load Solution	∼6 M HCl, trace HNO_3_	∼16 mL
1	6 M HCl	20 mL
2	4 M HCl – 0.1 M HF	25 mL
3	4 M HCl – 0.1 M HF	25 mL
4	2 M HNO_3_	20 mL
5	2 M HNO_3_	20 mL
6	2 M HNO_3_	20 mL
7	Conc. HNO_3_	10 mL
8	Conc. HNO_3_	10 mL

## 3 Results and discussion

### 3.1 Chemical separations

**FIGURE 6:**
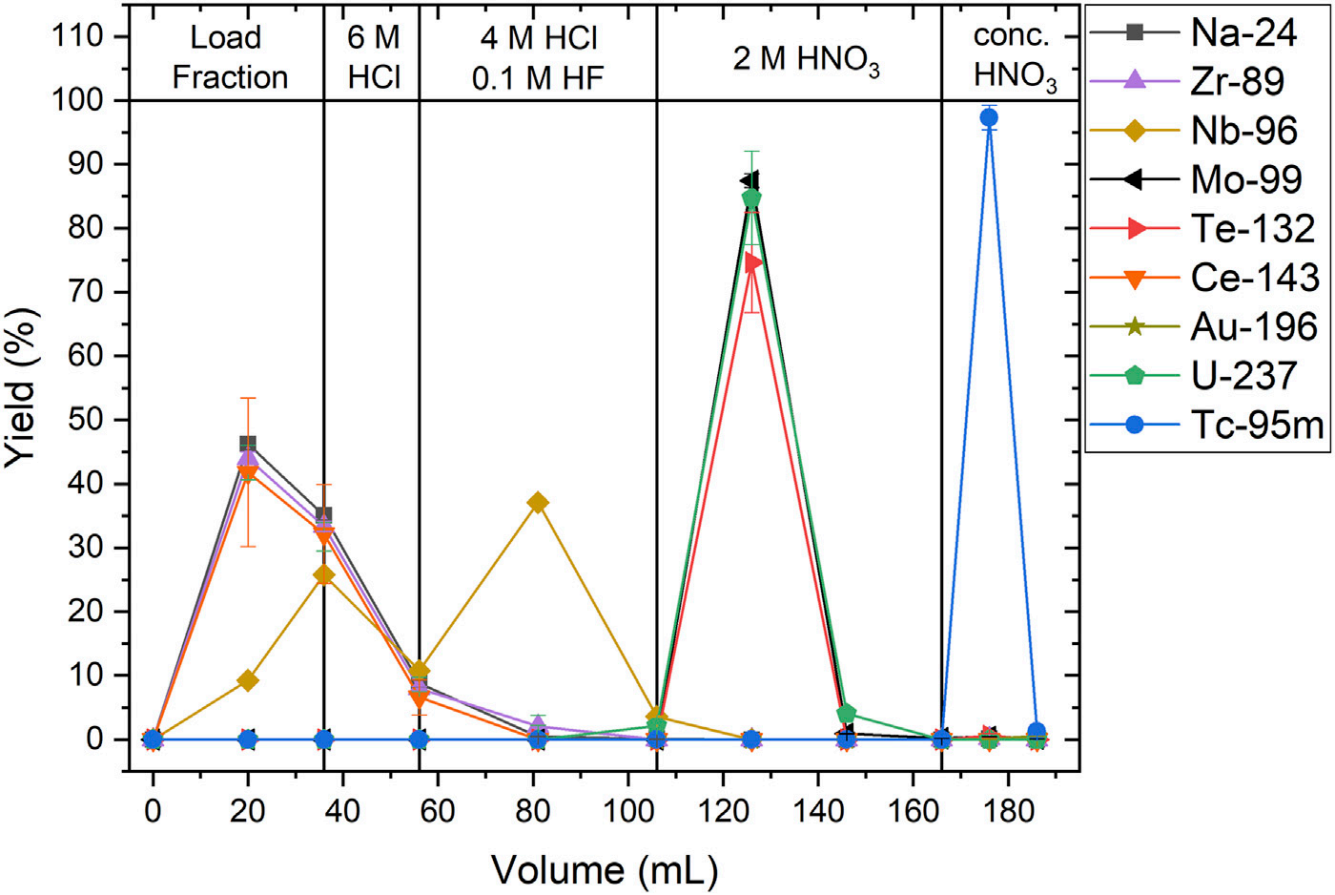
Typical elution profile for the SRC separation chemistry. Data from N250118.

The aim of the chemical separations is to separate Mo and Tc from the bulk SRC mass, from other radioisotopes, and separate Mo and Tc from each other. First, the leaching process is used to remove the upper layers of the SRC with the embedded debris. Leaching, rather than dissolution, limits the amount of bulk material that needs to be processed to ∼100–120 mg V per SRC rather than the whole SRC (∼6 g). An anion exchange column is then used for the separation of the isotopes of interest. A typical elution profile for the chemical separation is shown in Figure 6. While a wide range of fission products and activation products are produced in the experiment, only a limited selection of fission products and activation products have properties appropriate for detection after the chemical processing. For example, short lived fission products, such as ^92^Sr (2.61 h) and ^128^Sb (9.1 h), are detectable in the initial counts of the SRC (∼3 h post shot), but by the time the chemistry samples are counted (∼20 h post-shot) they have decayed to background levels. Furthermore, due to the high heat used for the leaching process, gaseous fission products (Xe, Kr, I) are volatilized during the leaching process and not reliably detected in the chemistry samples.

**FIGURE 7:**
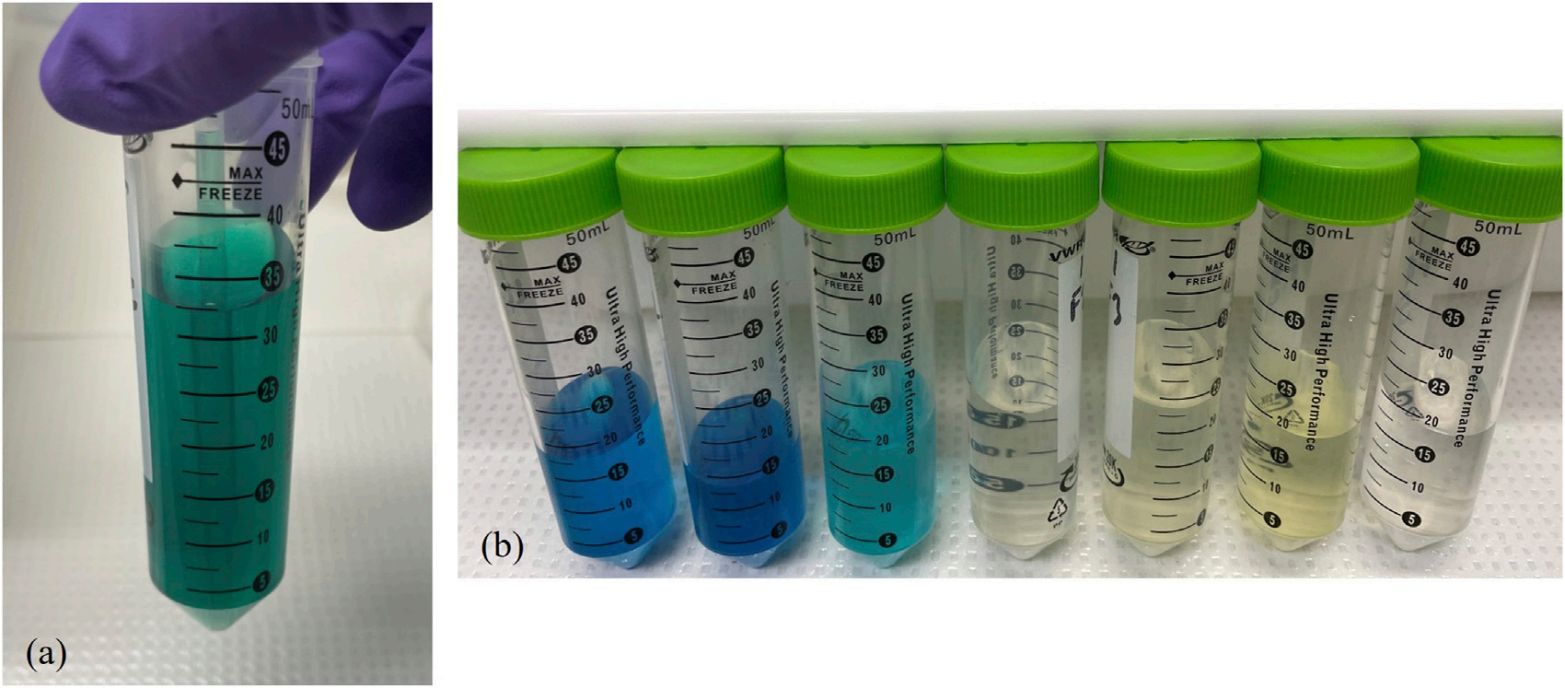
**(a)** Leaching solution after dilution with water to load onto column. **(b)** Initial fractions from the column separation, showing the elution of V.

After leaching, there is a mixture of V^4+^ (blue) and V^5+^ (yellow) creating a green solution (Figure 7A). The lower oxidation state, V^4+^ is eluted with the load solution and the initial washes with HCl solutions; V^5+^ is eluted with Mo and U in 2 M HNO_3_. This can be seen in Figure 7B, which shows the column fractions and their progression from blue to clear (elution of V^4+^) to yellow (V^5+^) and clear again.

Many other elements, like Na and Ce, that do not form anions in HCl also have no affinity for the resin and are removed with the V^4+^. Other notable fission products, specifically Zr and Nb, are removed with the HCl washes as well. The behavior of Zr is primarily driven by hydrolysis, it is not stable in 6 M HCl without HF and elutes immediately, likely in a hydrolyzed form. Niobium is also not stable against hydrolysis in 6 M HCl; this leads to the double humped distribution where the hydrolyzed species bleeds off the column in 6 M HCl, but it is not fully stripped until an HCl-HF solution is eluted. The 2 M HNO_3_ fractions elute Mo, U, and Te, all three of which form anions in HCl solutions but are neutral to cationic in HNO_3_. As ^237^U and ^132^Te are present at activity levels more than an order of magnitude lower than ^99^Mo and have low energy gamma-ray emissions that do not interfere with the detection of ^99^Mo, no further separation is required to remove them from the Mo fractions. Finally, Tc, likely complexed as the pertechnetate anion (TcO_4_^−^), is retained until elution with concentrated HNO_3_. Gold is retained on the resin and not effectively stripped with any acid concentration, though there is a small amount (0.6%) of ^196^Au breakthrough in the final HNO_3_ fractions.

Chemical yields for the main reaction products are shown in Table 3. All isotopes shown (apart from ^95m^Tc) are detectable in the initial SRCs as well as the chemistry samples, enabling the total chemical yield to be calculated. For each set of SRCs, the leaching yield for ^99^Mo can be determined by counting all chemistry fractions that contain ^99^Mo and comparing the collected activity to the initial SRC activity. The column yield for Mo is ∼100%; the column itself does not retain any detectable level of ^99^Mo after the chemistry is completed and transfer losses are negligible compared to the uncertainties from counting statistics. The leaching process has a yield of ∼85–95% ^99^Mo, and it varies slightly between experiments and is measured for every SRC to correct the final data for losses at this stage. This is likely due to the qualitative nature of the leaching process as it is based on a color change. Furthermore, as the SRC itself partially melts due to the heat from the implosion, the debris is mixed into the bulk V material rather than just collected on the surface. The melt depth is highly dependent on the neutron yield and laser input power, which varies between experiments and introduces another source of variability. It is assumed that the leaching yield for Tc is the same as for Mo due to the chemical similarities between these elements. As previously discussed, a chemical yield tracer is added to the column to determine the column yield for Tc.

**TABLE 3:** Overall chemical yields for major fission products and activation products. This data is from N250118 and representative of the data from other experiments. As ^95m^Tc is a tracer added to the samples after leaching, the yield represents only the column yield.

Isotope	Chemical processing yield (%)
^24^Na	91 ± 1
^89^Zr	88 ± 1
^96^Nb	87 ± 0.9
^99^Mo	89 ± 1
^132^Te	75 ± 8
^143^Ce	80 ± 1
^196^Au	0.61 ± 0.014
^237^U	91 ± 7
^95m^Tc	98 ± 2 (column yield)

**FIGURE 8:**
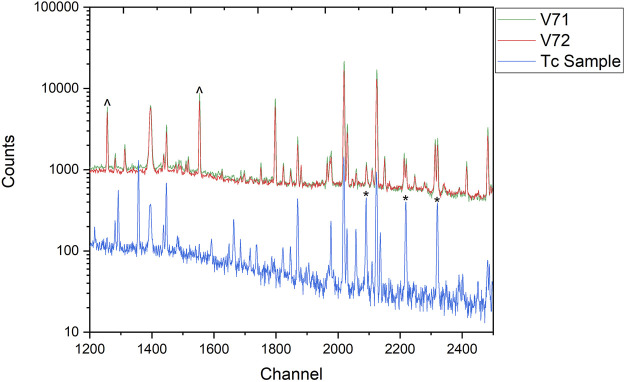
A comparison of two SRCs (V71 and V72) as compared to the final Tc sample, the product of chemically processing both SRCs. The energy region is approximately 445 keV–915 keV. The major peaks from ^95g^Tc (766 keV) and ^96g^Tc (813 keV, 850 keV) are indicated with asterisks. The particurly broad peak around channel 1,400 is the 511 keV annihilation line. Major peaks from ^96^Nb are indicated with carrots (^).

**FIGURE 9:**
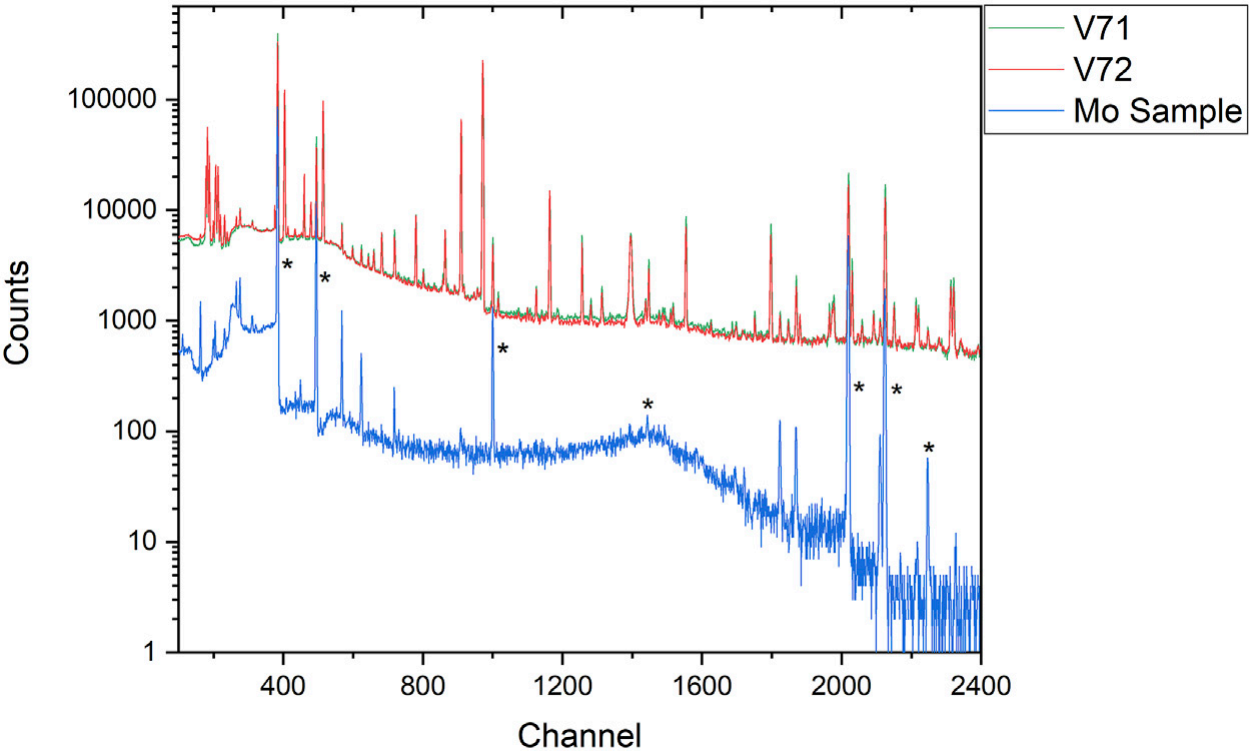
A comparison of two SRCs (V71 and V72) as compared to the first Mo fraction from the separation of the combined SRC leaching solutions. The energy region is approximately 40 keV–880 keV. Relevant peaks are indicated with asterisks; the lowest energy peak is from the IT decay of ^99m^Tc (the daughter of ^99^Mo), the remaining peaks are from the decay of ^99^Mo.

The removal of fission products is crucial as these radionuclides create a high background with numerous gamma-ray emissions that make it impossible to detect the low activity Tc isotopes. This is shown in Figure 8, which compares the gamma spectrum of two SRCs before chemical processing as compared to the Tc sample from the processing, and combining, of both SRCs. The chemical separations significantly reduce the background, allowing the gamma-ray emissions from ^95g^Tc and ^96g^Tc, largely in the 750–850 keV range, to be detected with improved resolution and uncertainty. A similar comparison for Mo is shown in Figure 9.

The separation procedure is highly effective at removing fission products and activation products from Mo and Tc. In particular, the chemical separation has a high separation factor for Tc/Nb which is critical as ^95^Nb is a fission product which only has one significant gamma-ray emission at as 765.8 keV, exactly the same as ^95g^Tc. Therefore, these two isotopes cannot be differentiated solely based on gamma spectroscopy. Fortunately, ^96^Nb, another fission product, has numerous gamma-ray emissions and is present at higher activity levels than ^95^Nb (∼10x), providing a Nb tracer that can be used unambiguously to determine if Nb is present the samples. In Figure 8, two major gamma-ray emissions from ^96^Nb are indicated with carrots (^), these are indistinguishable from the background in the Tc spectrum. As the activity of ^95^Nb is an order of magnitude lower than ^96^Nb, this potential interference from ^95^Nb does not affect the final reported ratio.

The overall chemical processing, leaching, column separation and counting sample preparation, can be completed in ∼7 h with high yields for Mo and Tc (>80%). The rapid timeframe is critical for the determination of ^95g^Tc activity levels due to its short half-life. This is possible as the chemical processing has been optimized to ensure only one column separation is required to achieve sufficient radiopurity and no evaporation steps are needed. The procedure does not require specialized equipment other than the leaching cells, which are custom made at LLNL, making it feasible to use this process as a routine diagnostic for the PSS campaign.

### 3.2 Results from PSS Mo capsule experiments

**FIGURE 10:**
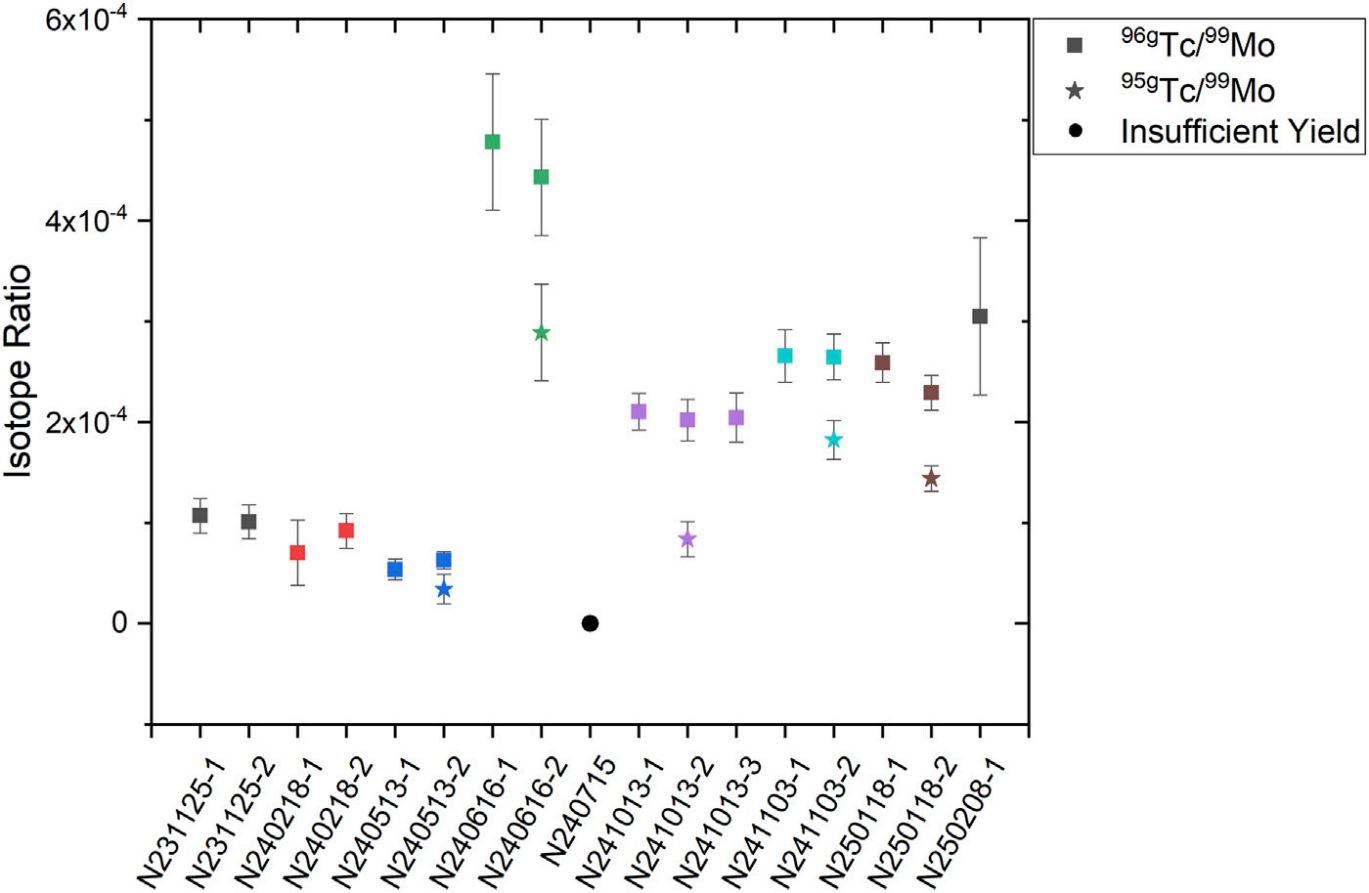
Measured isotope ratios (^96g^Tc/^99^Mo, ^95g^Tc/^99^Mo) from all PSS Mo capsule experiments with radiochemical diagnostics. Experiments are indicated by the date (NYYMMDD) and a number (−1, 2, 3) to indicate the sample number from that experiment. Independent measurements from the same NIF shot are shown in the same color.

The results from the radiochemical analysis for PSS Mo capsule experiments from November 2023 to date (February 2025) are shown in Figure 10. The ratios are calculated from the number of atoms of the Tc isotope of interest (either ^95g^Tc or ^96g^Tc) divided by the number of atoms of ^99^Mo. The number of atoms of ^99^Mo is known directly from the initial counts of the SRC. The number of Tc atoms, of each isotope, is assessed from the chemistry samples and corrected for the leaching yield (as determined from the initial counts of the SRC) and the chemical yield (as determined by the Tc tracer). The uncertainty on the ratio arises from propagating the statistical uncertainty from nuclear decay counting with the systematic uncertainties from the chemical processing (i.e., the errors on the chemical yield and leaching yield) and the detector calibration.

As discussed previously, usually four SRCs are fielded on 90–315 leading to two independent measurements for each experiment (as the SRCs are combined in sets of two during the chemical processing). Occasionally, additional SRCs can be fielded on 90–78, which allows for a third independent measurement from two SRCs. This was performed on N241013 as a trial to measure the ^96g^Tc/^99^Mo ratio from two SRCs from 90–78, as well as the standard four SRCs from 90–315. Efforts to routinely increase the number of measurements of both ^96g^Tc and ^95g^Tc per shot are underway.

While there are differences between the various experiments, measurements of the same ratio from the same experiment have always been within error, indicating there are no large geometric effects or systematic errors that might be affecting the SRC collection. This was particularly crucial to assess for N241013 as data was collected from both 90–315 and 90–78, with a much different line-of-sight, and the ratios measured from SRCs from both positions were in agreement within error. More data from both positions would be required to definitely conclude that the isotopic ratios are line-of-sight independent, but these results indicate that it may be, which would align with predictions based on the production of these isotopes and the known conditions of the implosion. The notably high outlier (N240616, green) is due to a different fuel composition. In this experiment, the capsule was filled with a mix of hydrogen, deuterium and tritium, rather than a 50–50 mix of deuterium and tritium as for all the other experiments. Hydrogen gas leads to an increase in available protons for (p,xn) reactions, and therefore more Tc isotope production, as protons are a component of the fuel rather than just a byproduct of knock-on reactions in DT fuel or DD fusion.

In general, a higher ratio should indicate more mix given identical fuel conditions. However, as discussed in (MacLaren et al., 2025), the measured ratios are not directly used to assign a value to the amount of mix, but are used to benchmark codes that model the experiments parameters. Each experiment is fundamentally different from the other experiments as the capsules are not uniform (due to manufacturing challenges), the fuel ratios and amount change between experiments, and the laser power is not constant due to facility priorities and maintenance schedules. Therefore, the mix ratio needs to be taken in context with the other experiment parameters through detailed models that incorporate capsule irregularities and the laser conditions. As more experiments are performed, replicate experiments may be completed, in which case statistical analysis of the measured ratios may be meaningful.

Neutron yields for these experiments are listed in Table 4. The minimum yield to produce sufficient activities of ^99^Mo and ^96g,95g^Tc for analysis is ∼1 × 10^14^ neutrons. The effect of low yields can be seen in Figure 10 with N240616 and N250208, both of which have higher error bars due to low counting statistics. However, they can still be directly compared to the other experiments because reporting a ratio, rather than absolute activities, normalizes for yield as the atoms of ^99^Mo is directly proportional to the yield as described in the Introduction. This allows the amount of capsule mix to be assessed for a variety of different capsule designs as well. For example, N231125 had 6 mg/cc of DT fuel while N240218 had 9 mg/cc. The higher fuel density did not significantly change the yield nor induce significant changes in mix, which is valuable data as increased mix can decrease yield but can be ruled out as a cause in this case. Similarly, N241013 and N250118 had 6 mg/cc and 9 mg/cc, respectively, of DT fuel but no anti-mix layer (a thin layer of Be without Mo between the fuel and the Be-Mo gradient) unlike the earlier shots (N231125 and N240218) leading to higher mix ratios.

**TABLE 4:** Neutron yields for PSS shots with radiochemical separations performed to diagnose capsule mix.

NIF shot	Neutron yield (×10^14^)
N231125	1.09 ×10^15^
N240218	7.58 ×10^14^
N240513	9.58 ×10^14^
N240616	2.36 ×10^14^
N240715	1.44 ×10^12^
N241013	1.42 ×10^15^
N241103	8.11 ×10^14^
N250118	1.64 ×10^15^
N250208	1.84 ×10^14^

### 3.3 Contribution of ^99^Mo from DU fission

As ^99^Mo is a high yield fission product, it was necessary to determine if there was an interference with the measurement of ^99^Mo production from the capsule versus ^99^Mo production from fission in the depleted U hohlraum material. Any ^99^Mo from DU fission would artificially lower the Tc/Mo ratio used to assess the mix and introduce systematic uncertainties. To address this, measurements of ^99^Mo were performed on SRCs from a range of experiments, including those with Mo in the capsule and designs with no Mo in the target assembly (where ^99^Mo would be produced exclusively from fission). To enable direct comparison of results from different experimental campaigns at NIF, only the initial counting data (prior to any chemical processing) was used for this assessment since not all SRCs from other campaigns underwent chemical processing.

**FIGURE 11:**
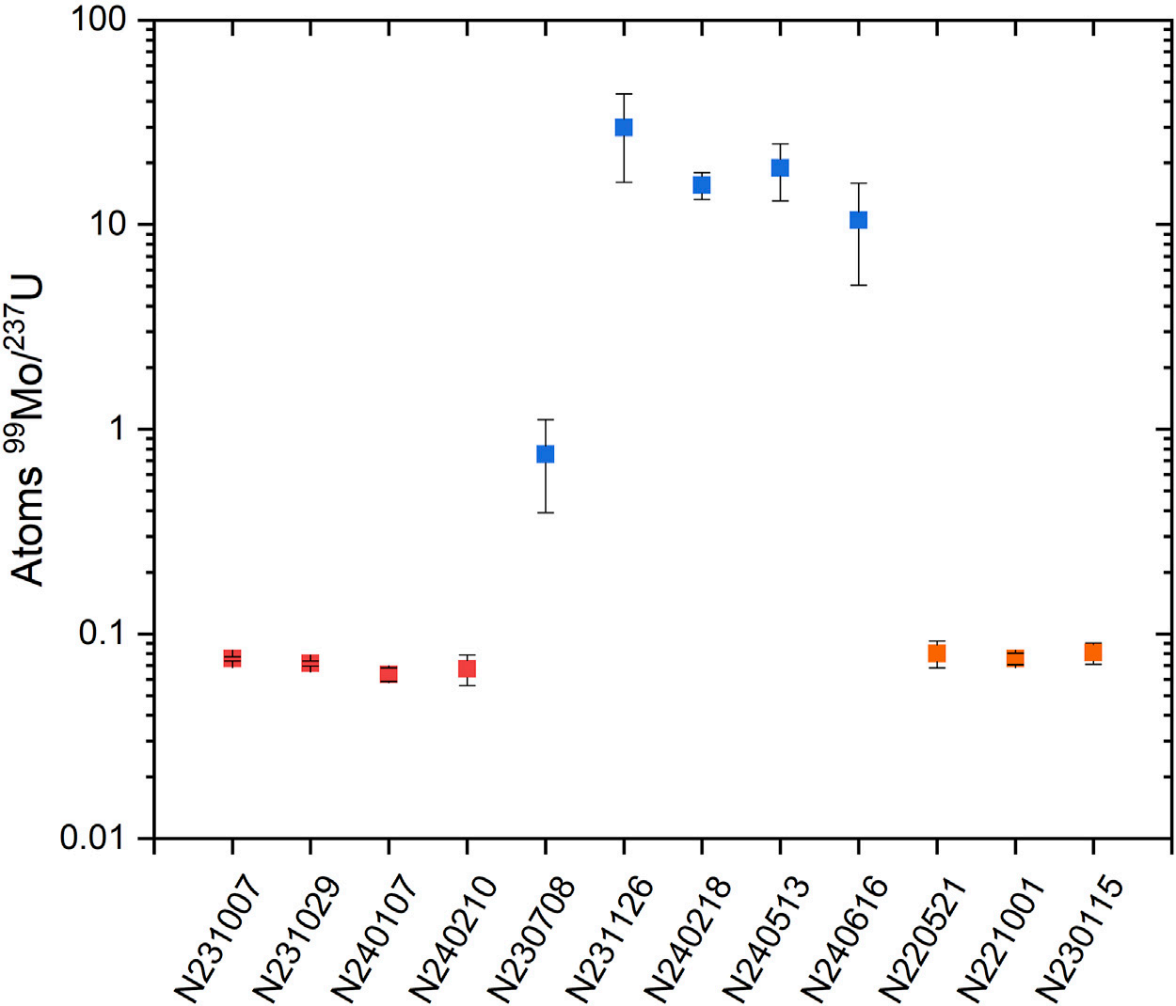
A comparison of ^99^Mo production across different experimental campaigns at NIF. Hybrid-E experiments are shown in red, PSS Mo capsules in blue and PSS Cr capsules in orange.

A plot of the ratio of atoms of ^99^Mo to the number of atoms of ^237^U for three different capsule designs, all with Au-lined DU hohlraums, is shown in Figure 11. The value is determined from dividing the number of atoms of ^99^Mo by the number of atoms of ^237^U from the direct counts of the SRCs for these experiments. The uncertainty is from counting statistics as well as detector calibration. As with the Tc/Mo ratios, using a ratio rather than an absolute number normalizes for the yield and allows for comparison between experiments with different yields. In this case, the production of ^237^U via the ^238^U (n,2n) reaction scales the neutron yield and it is produced in every experiment with a DU hohlraum. The first set of experiments shown in Figure 11 (red) are from the Hybrid-E campaign, a design without Mo in the capsule. This campaign uses high density carbon capsules doped with tungsten and has produced neutron yields of >4 × 10^17^, the highest ever achieved at NIF and at least two orders of magnitude greater than PSS Mo capsule yields. The second set of experiments (blue) are PSS Mo capsule experiments. The final set are PSS Cr capsule experiments (orange). Even in cases with extremely high yields, and proportionally more fission, the ratio of ^99^Mo/^237^U is orders of magnitude lower for shots without Mo in the capsule as compared to the PSS Mo capsule experiments. This demonstrates that the production of ^99^Mo from the capsule is more than an order of magnitude higher than the ^99^Mo produced via fission when normalized for the yield. Therefore, interference from the production of ^99^Mo from fission is negligible for the PSS experiments compared to the production of ^99^Mo in the capsule, and no corrections are required to utilize the Tc/Mo ratios as an indicator of mix.

### 3.4 Effect of metastable states of ^96^Tc and ^95^Tc

Both Tc isotopes of interest, ^95g^Tc and ^96g^Tc, have a metastable state with a significant half-life. The timeline for measurements precludes the direct detection of ^96m^Tc (52 m) as the counting for the purified Tc isotopes does not begin until ∼20 h post-shot. Any ^96m^Tc produced in the experiment would have decayed by the measurement time, primarily to ^96g^Tc via isomeric transition (IT) decay (∼98%) (NNDC, 2023). Therefore, the measured ^96g^Tc is interpreted as the cumulative production of both the metastable and ground state.

**FIGURE 12:**
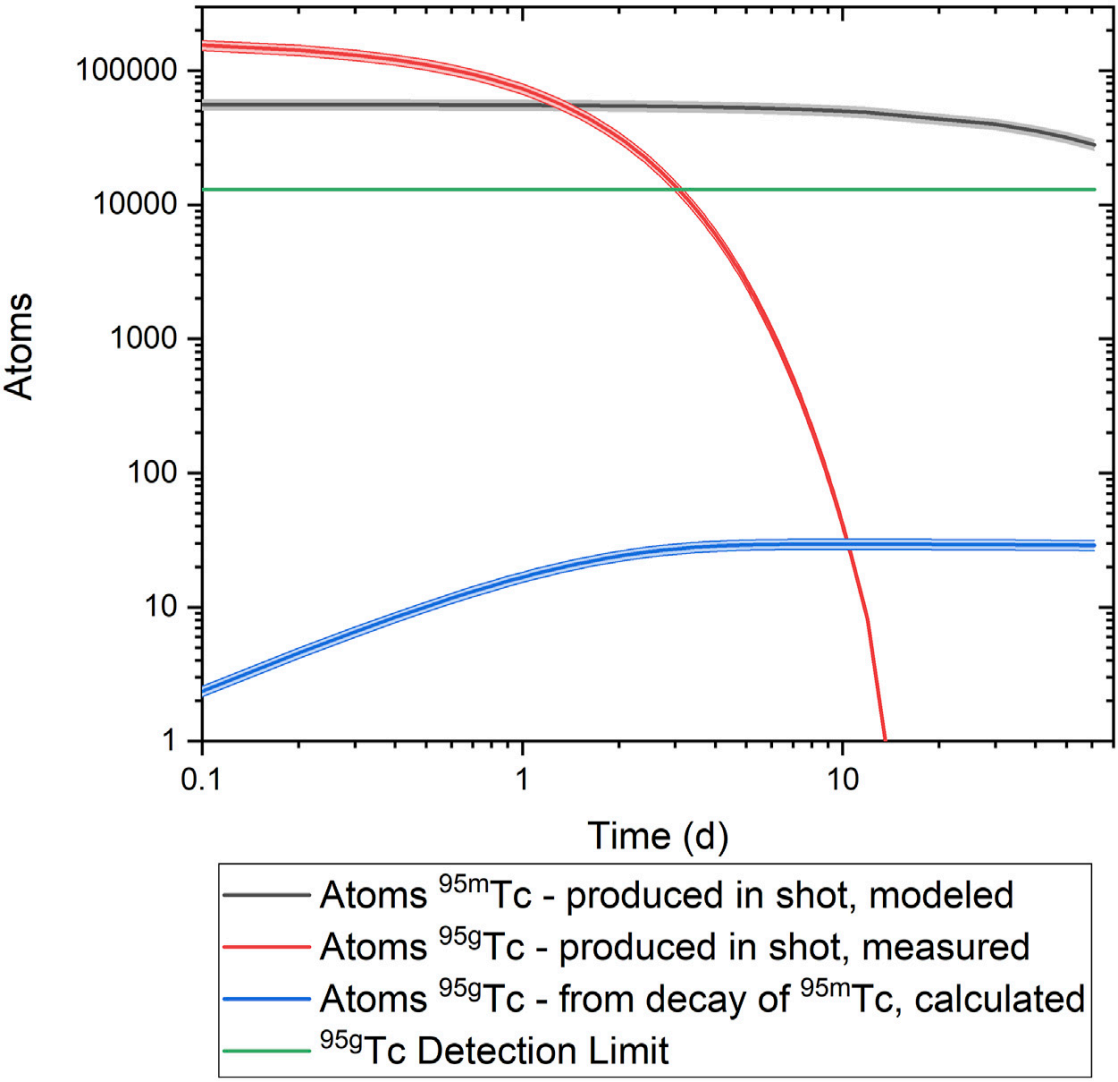
The decay of ^95g^Tc as measured from the chemistry samples compared to the calculated activity of ^95m^Tc and the resulting in-growth of ^95g^Tc from the IT decay.

The situation for ^95^Tc is more complicated as the metastable state is longer-lived than the ground state and comes into secular equilibrium with the shorter-lived ground state “daughter”, as briefly mentioned previously regarding the tracer solution used for these experiments. Therefore, ^95g^Tc could be produced in the capsule as well as grow in continually from any co-produced ^95m^Tc. However, decay analysis is sufficient to demonstrate that the ^95g^Tc detected post-chemical processing is primarily from capsule production rather than in-growth from ^95m^Tc. Based on the reaction cross section network, models have indicated the production of ^95m^Tc should be about one-third that of ^95g^Tc. Based on these models, the theoretical number of atoms of ^95m^Tc and its decay is plotted in Figure 12. Due to the relatively low IT branch (3.88%), there is no significant in-growth of the ground state as compared to the detection limit. Therefore, the ^95g^Tc that is measured is produced from the shot and any effect from in-growth from the metastable state is negligible. Even in a worst-case scenario where the models are incorrect and there is 10x the production of the metastable state as compared to the ground state, there would still be insufficient impact from the in-growth of the ground state to alter the measurement as the resulting in-growth would still be an order of magnitude below the detection limit.

## 4 Conclusion

Radiochemistry is a valuable diagnostic for characterizing NIF implosions as it provides a route to directly assess the amount of fuel-ablator mix in an ICF experiment. This is a crucial parameter to assess when designing ICF implosions as the mix from the capsule into the fuel reduces both the compression and temperature of the DT fuel, lowering the final yield. Radiochemical signatures provide a unique diagnostic to inform these studies and complement other data, such as neutron yields, core ion temperatures, core neutron images and x-ray radiography, as they provide information about the capsule-fuel interactions in the burning plasma. Furthermore, as most NIF diagnostics are line-of-sight dependent and may have results impacted by the shape of the implosion or other factors that vary between shots, radiochemical results are independent of other diagnostics and likely have no geometric fractionation, although more data is required to verify this.

Under the current PSS campaign, measurements of the ^95g^Tc/^99^Mo and ^96g^Tc/^99^Mo ratios are an important factor to understand mix in these capsule designs. Establishing methods to measure these ratios, as demonstrated in this work, is a critical component of a long-running series of experiments utilizing Mo-based capsules. While the chemical methods used to quantify Tc and Mo are not applicable to other capsule designs, this work not only provides valuable data for the PSS campaign but serves as a proof-of-concept for establishing the use of radiochemical signatures as a mix diagnostic, which can be modified for future ICF experiments.

As new campaigns and new capsule designs are launched at NIF, the radiochemical signatures used to diagnose mix need to be adjusted as well. This requires consideration of extensive reaction networks and detailed calculations based on expected plasma conditions to determine which isotopes are expected to be produced in detectable quantities. Then new chemical methods must be developed to target the isotopes of interest and separate them with sufficient purities and within a reasonable timeframe based on the half-lives. This development work occurs in the year or years prior to the first capsule experiments and requires integration of chemistry, physics and computer science. Future work in this area is already underway to develop a radiochemical diagnostic for tungsten, used in the Hybrid-E capsules (Zylstra et al., 2022), and its reaction product, rhenium.

The integration of radiochemical results with detailed simulations of capsule performance and plasma conditions allows for the optimization of factors such as capsule configuration, laser power and fuel density to improve yields and further the study of fusion science at NIF. These experiments are expected to continue for many years and be extended to other NIF campaigns beyond PSS to provide data on a range of capsule designs and experimental platforms.

## Data Availability

The original contributions presented in the study are included in the article/supplementary material, further inquiries can be directed to the corresponding author.

## Author disclaimer

This document was prepared as an account of work sponsored by an agency of the United States government. Neither the United States government nor Lawrence Livermore National Security, LLC, nor any of their employees make any warranty, expressed or implied, or assume any legal liability or responsibility for the accuracy, completeness, or usefulness of any information, apparatus, product, or process disclosed, or represent that its use would not infringe privately owned rights. Reference herein to any specific commercial product, process, or service by trade name, trademark, manufacturer, or otherwise does not necessarily constitute or imply its endorsement, recommendation, or favoring by the United States government or Lawrence Livermore National Security, LLC. The views and opinions of authors expressed herein do not necessarily state or reflect those of the United States government or Lawrence Livermore National Security, LLC, and shall not be used for advertising or product endorsement purposes.
